# Work characteristics, motivational orientations, psychological work ability and job mobility intentions of older workers

**DOI:** 10.1371/journal.pone.0195973

**Published:** 2018-04-27

**Authors:** Carlos-María Alcover, Gabriela Topa

**Affiliations:** 1 Department of Medicine and Surgery, Psychology, Preventive Medicine and Public Health, Immunology and Medical Microbiology, Nursing, and Stomatology, Faculty of Health Sciences, Universidad Rey Juan Carlos, Alcorcón, Madrid, Spain; 2 Department of Social and Organizational Psychology, Faculty of Psychology, Spanish University for Distance Teaching, Madrid, Spain; IUMPA - Universitat Politecnica de Valencia, SPAIN

## Abstract

Drawing on job design theories and a conceptual framework of work-related goals and motivation in later adulthood, the aim of this paper is to explore how work-related and individual factors are separately and jointly related to psychological work ability and bridge employment intentions via late job mobility. The cross-sectional study is based on a sample of 171 older Spanish workers aged 45–65 and beyond. We differentiated between groups of older workers in mid career (45–55 years of age) and in their later careers (56 years and beyond). Our results confirm that task characteristics and, secondarily, knowledge characteristics are the most relevant factors in perceptions of psychological work ability among aged workers. Both age groups display a very marked personal mastery trait, which mediates the relationships between job characteristics and both psychological work ability and late job mobility intentions. The paper concludes with a discussion of theoretical and practical implications and suggestions for future research on the issues implied in the psychological adjustment of older workers in their mid and late careers.

## Introduction

The workers of the baby boom cohort (i.e. those currently aged 46–65) have seen major changes in their work context over the last decade. The rise of the knowledge-based job has raised cognitive and psychosocial prerequisites while lowering physical demands, and technological innovations have intensified the need for recycling and continuous learning to acquire new skills and competences [1]. In addition to conventional task-related requirements like autonomy, variety and feedback, the characteristics of work in these new scenarios include increasing knowledge-related demands (complexity, data processing and problem solving) and social requirements (interdependence, social support and, interaction outside the organization) [2].

The ways in which older workers experience and deal with these new scenarios and challenges will determine not only their psychological adjustment and their work ability, which is to say the relationship and balance between their personal resources and the demands of the job [3, 4] in the middle and late stages of their careers, but also their retirement intentions and desire to continue working [5, 6].

From an organizational and human resources management perspective, older workers already play a critical role in meeting workforce needs, and they will continue to do so for years to come. In this light, it is crucial for organizations to design and implement flexible strategies to retain skilled and motivated older workers [7], as well as recruitment policies and processes to accommodate an older workforce [8, 9].

In these news scenarios it is critical to examine the motivational orientations of aging workers [10, 11]. While this group has attracted considerable research interest in recent years [12], psychological issues have often been overshadowed by a focus on physical and financial questions [6, 13]. In this light, it has become necessary to broaden our understanding of the motivational factors affecting career decisions in the mid and late stages of working life.

The objectives of this study are threefold. First, we aim to improve our understanding of the different dimensions of job characteristics (task-related, social, and knowledge-based characteristics) and their relationships with psychological perceptions of work ability and late job mobility intentions. Second, we explore the moderating role of workers’ age on the direct relationships between job characteristics and work ability and job mobility intentions. Finally, we explore the mediating role of older workers’ motivational orientations in the relationship between job characteristics and work ability and job mobility intentions. Our aim, then, is to shed light on the work context and motivational factors affecting the psychological adjustment of workers who have entered the middle and later stages of their professional lives.

### Work ability and job mobility intentions in older workers

Work ability has been defined as the combined result of physical abilities and cognitive faculties as assessed in relation to task demands, whether intellectual or physical [14]—in other words, it is a product of both the individual and the working environment [15].

Prior work ability research has found empirical support for many individual and work-related correlates of work ability, including physical and psychological work demands [14, 16, 17], work resources including autonomy, developmental opportunities and supervisor support [14, 16], and psychosocial factors such as perceived work attitude, styles of coping and perceived organizational support [17, 18]. A recent model [19] defines perceived work ability as an individual’s self-perception or evaluation of his or her ability to continue working in his or her job. Perceived work ability stems from an individual’s experience related to a number of work factors, along with the degree to which he or she possesses personal resources that facilitate positive work ability perceptions, including personal resources (positive affectivity and emotional stability, among others). To date, however, the proposed models on work ability have not included motivational factors, such as motivational orientations. Our study attempts to fill this gap taking motivational orientations as the mediator variable between perceived job characteristics and work ability.

Work ability is primarily a question of balance between work demands (physical and/or psychological) and personal resources [20]. Moreover, research shows that the number of physically demanding jobs has shrunk in the USA to the point where they occupy only around 7% of the workforce [21]. Few studies have investigated work ability in occupational populations with predominantly mental demands at work, and our understanding of psychological work ability remains very sketchy [22]. Given that both personal resources and work demands usually change with age, this study will address psychological work ability via the expertise and knowledge held by older workers, focusing in particular on cognitively demanding jobs.

Besides work ability, research can also examine stability and change in adjustment processes at work of older employees through objective and subjective factors [23], such as work engagement and motivation, work performance or active work participation as indicators of positive adaptation to job and the maintenance of work ability [24]. Thus, continued employment participation (e.g., bridge employment) might be the ultimate criterion for successful psychological adjustment to employment in late career [23].

The concept of bridge employment refers to a whole range of different possible work situations [25] but it is most commonly defined as any kind of paid work (part-time, full-time, or self-employment) carried on after the end of an individual’s professional career or full-time employment but before complete withdrawal from the labor force or retirement [26, 27]. Bridge employment alternatives may therefore be considered modes of retirement that prolong working life, allowing the term “full retirement” to be used to refer to final withdrawal from the workforce [28]. The transitions characterizing bridge employment occur both within the individual’s own profession and in other occupations, and they can take the form of (full- or part-time) wage-and-salary employment, permanent or temporary jobs and self-employment [29, 30].

A job-type change or job mobility is a bridge employment decision that entails a greater degree of initiative and potential risks than other modalities, such as prolonging working life in the same job or organization [31]. A job-type change can be considered a *career change* or “entry into a new occupation which requires fundamentally different skills, daily routines, and work environments from the present on” [32]. Hence, such changes may include either full- or part-time work, as well as temporary employment, of a kind that offers an eventual bridge to full-time retirement [27]. Since job mobility has been traditionally the most limited bridge employment modality in post-career employment [33, 34], we consider relevant to know the intentions of older workers regarding this option.

### Work characteristics

The term “work design” is used to describe the ways in which jobs, tasks and roles are structured, linked together and changed, as well as the impacts which such structures, interconnections and changes have on individual, group and organizational outcomes [35]. The nature of the task itself has conventionally been seen as the key factor affecting the outcomes obtained in work design implementations. However, other social and structural influences may also be discerned [36] when the *situational* and *social* context of tasks is considered [37]. The literature identifies a range of models and instruments in this regard. Perhaps the most widely accepted and used concept in the last forty years has been that of motivational work design characteristics (e.g. [38]). Nevertheless, the validity of this model has been questioned in recent years, because it focuses only on the motivational features of the job itself while ignoring other aspects like social and contextual characteristics. Furthermore, uncritical acceptance has prevented rigorous theoretical development and hindered the progress of our knowledge in this area [2].

Existing empirical studies have conclusively shown that job characteristics are related to a range of personal and organizational outcomes [36, 38]. Meanwhile, task and knowledge characteristics should affect a broad range of workers’ attitudes and behaviors because they refer to the ways in which work is done. Finally, social characteristics, which include interdependence and social support, involve the interplay of tasks and role enactment, and hence they should in turn affect worker outcomes. Given the comprehensive nature of WDQ [39] (a work design measure that identifies four main factors, each of which embraces various characteristics, as we describe in the "Methods" section), our study will focus on task, knowledge and social characteristics. Previous research shows that the occupation-level factor that most motivates older workers to enter new careers is probably the degree of change in task, knowledge and social skills [31]. Following Truxillo, Cadiz, Rineer, Zaniboni and Fraccaroli [40], then, we will not include context characteristics (ergonomics, physical demands, equipment use and work conditions), “which are well established to be affected by workers age” (p. 344), and have already been thoroughly analyzed in prior research.

### Moderating role of age

According to the data, the number of older workers remaining in the labor market in jobs that do not entail significant physical demands has increased significantly over the last two decades [41, 42], which would show *prima facie* that older workers are able successfully to cope with the cognitive, emotional and relational demands of their work. For instance, the results of a meta-analysis conducted by Sturman [43] concluded that, over time, experience becomes more predictive of job performance in high complexity jobs. This implies that older employees may be able to compensate to some degree for cognitive changes in a manner that does not automatically result in poor performance [44].

Meanwhile, various studies examine how older workers address the different characteristics of their jobs and how such features interact with other motivational factors. For example, Zacher and Frese [45] showed that the interaction between age and task complexity affects a motivation outcome, which they called “perceived opportunities at work”. Similarly, Zaniboni, Truxillo, Fraccaroli, McCune and Bertolino [46] found that age moderated the relationships between job characteristics and workers’ satisfaction. Hence, it seems reasonable to expect that workers may react in different ways to the job characteristics in the mid and late stages of their careers, and this in turn would affect their work ability perceptions.

Based on the constructs described above and the possible relationships existing between them, we propose a series of hypotheses to explore their interaction in a sample of workers who have reached the mid or late stages of their careers. Our study is designed to allow joint analysis of job-level variables (work characteristics) and individual conditions (motivation), two out of the three factors which are believed to impact the mid and late career stages [47], considering age differences in relation to psychological work ability and job mobility intentions as outcomes.

The concept of age is of course multidimensional [48, 49]. However, chronological age has conventionally been the most widely used indicator used both in research and in the design of organizational policies, which seems reasonable, given that it can be easily measured, is objective and affects everybody, and although it exhibits covariance with other personal characteristics like cognitive capacity, health and subjective age, these factors are themselves hardly separable from age itself. Therefore, this study follows Truxillo et al. [40] in using chronological age as an effective observable indicator for research purposes and for human resources management decisions [50]. A common suggestion in the literature is that middle-aged and older workers range from 40 to 70 years [51]. And most researchers in the field of work and aging refer to older workers as between 55 and 70 years of age [52]. Based on these rationales, we formulated the following hypotheses.

*Hypothesis 1*: age moderates the direct relationship between work characteristics (i.e. task (*H1a*), knowledge (*H1b*) and social (*H1c*) characteristics) and psychological work ability. In all cases, therefore, the direct, positive effect of work characteristics on psychological work ability will be more intense when the worker is below 55 years of age and less intense when the worker is older than 56 years.

*Hypothesis 2*: age moderates the direct relationship between work characteristics (i.e. task (*H2a*), knowledge (*H2b*) and social (*H2c*) characteristics) and job mobility intentions. In all cases, therefore, the direct, positive effect of work characteristics on Job mobility Intention will be more intense when the worker is below 55 years of age and less intense when the worker is older than 56 years.

### Work motivation in older workers: Mediating role

No published studies report significant differences in motivation level between different age groups, which means that older workers are not less motivated than their younger colleagues, despite what age stereotyping might suggest [5]. Some studies even show that older workers are highly motivated [53], although the results of this research point to differences between age groups in the factors which explain motivation [12, 54–56].

As a consequence, a number of scholars have recently argued that our conceptualization of work-related motives is in need of reformulation from a lifespan perspective, as research findings consistently reveal differences in the predominant motives between age groups. Kooij et al. [12] performed a meta-analysis of the associations between age and the five basic motives (intrinsic, extrinsic, growth, social and security motives), which revealed a significant positive relationship between age and intrinsic motives, and a significant negative relationship between age and the strength of growth and extrinsic motives. Furthermore, the predicted positive relationship between age and the strength of social and security motives was only found in certain occupations [12]. The authors go on to argue for the development of tools to measure emergent motives like generativity, knowledge utilization, helping, collaboration, and enhancing positive affects [57], and they end with a tentative exploration of the potential relationships between these measures of motives and age.

Based on their comprehensive review of the latest theoretical developments and leading edge research into work motivation, psychosocial research suggests that it may be possible to conceptualize differences in achievement motivation in terms of individual differences in goals, and the majority of researchers concur in distinguishing between appetitive (approach) and aversive (avoidance) motivational orientations [54]. Accordingly, Kanfer and Heggestad [58] proposed a developmental theory which distinguishes between distal influences on action (i.e. relatively stable motivational orientations), and proximal influences on performance linked to individual differences in self-regulatory, or motivational skills [54]. These authors also stress the importance of identifying individual differences in terms of competitive excellence motives and in aversively oriented motivational orientations like worry and emotionality with respect to performance demands [54, 58].

Building on these theoretical foundations, Heggestad and Kanfer [59] performed a series of empirical studies with the aim of developing a multiple trait motivational inventory explicitly designed to capture differences in motivational orientations. Based on their results, three basic factors may be identified, namely personal mastery, competitive excellence, and motivation-related anxiety. The results of studies carried out using this measure have shed considerable light on the motivation of older workers [54], and it has proved a very useful tool for research based on a holistic, worker-centered approach seeking to delineate the nature of older worker goals, their relationships over time, and the factors that influence motivation for goal accomplishments in later adulthood [11].

In line with Truxillo et al. [40], Kanfer and Ackermans’ work motivation framework [57] proposes different patterns of development that could throw light on the ways in which age and job characteristics interact to affect workers’ outcomes. Alternatively, by focusing on loss, growth, reorganization and exchange, we may be able to understand how older workers are able to adapt to tasks, craft their jobs and/or choose roles that better fit their strengths. To sum up, work motivation may play a mediating role between job characteristics and workers’ outcomes. Based on this rational, we formulated the following hypotheses.

*Hypothesis 3*: motivational factors (personal mastery, competitive excellence and motivation anxiety) mediate the direct relationship between work characteristics (i.e. task (*H3a*), knowledge (*H3b*) and social (*H3c*) characteristics) and psychological work ability.

*Hypothesis 4*: motivational factors (personal mastery, competitive excellence and motivation anxiety) mediate the direct relationship between work characteristics (i.e. task (*H4a*), knowledge (*H4b*) and social (*H4c*) characteristics) and job mobility intention.

**Fig 1:**
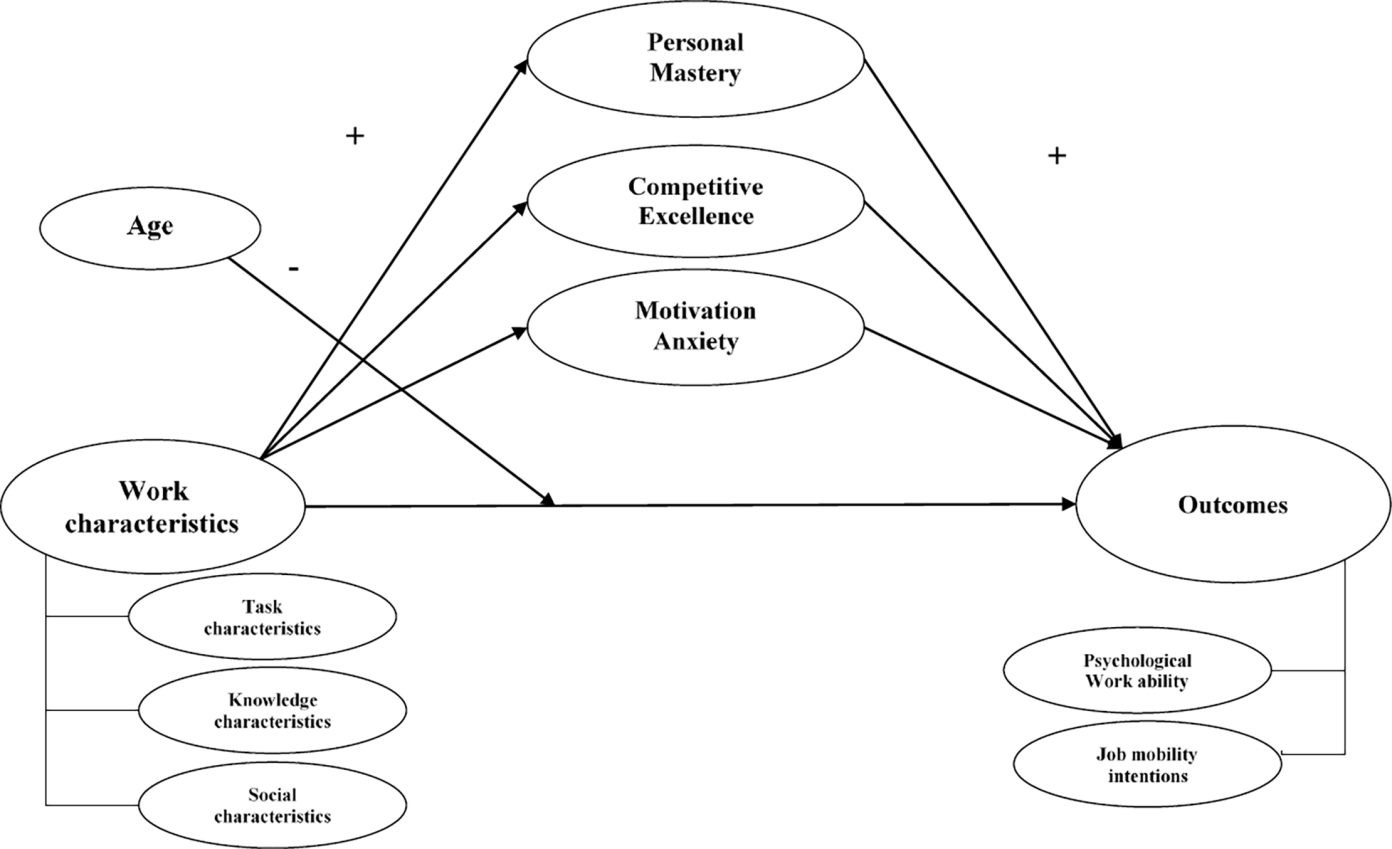
Theoretical model for the study.

To sum up, our study is designed to allow joint analysis of individual-level factors (motivation and age) and job-level factors (work characteristics), two of the three factors which are believed to impact the mid and late career stages [47]. Our approach therefore assumes that age moderates the relationship between perceived characteristics of work in three dimensions (task, knowledge and social dimensions) and perceived psychological work ability, and that motivational orientations mediate the relationship between work characteristics and perceived psychological work ability and job mobility intentions among groups of older workers. In this regard, we distinguish between mid-career workers aged between 45 and 55, and late-career workers aged over 56 years. Fig 1 below shows the final hypothetical model for our study.

## Methods

### Ethics statement

According to the certificate signed by the Secretary of the Ethics Committee in Research of the Universidad Rey Juan Carlos (Madrid, Spain), this study does not require any certificate from the ethics committee, given the nature of such research.

All the surveys analyzed in the study were voluntary completed, anonymous and dissociated and no personal information or data is recognized. It is in accordance with what is stated in the second paragraph, point 5 (Order SAS/3470/2009 December 16, Spanish Ministry of Health and Social Policy) and not being within the assumptions established in Article 2.e (Law 14/2007, June 3, Spanish Biomedical Research Law) concerning Biomedical research.

### Participants and procedure

The study was performed using a sample of older workers (N = 171) employed at various public and private organizations in the healthcare, financial services and consulting sectors. All the participants performed tasks of high qualification in their occupational sector (physicians, executives and senior consultants). Consequently, their tasks comprised the three work characteristics selected for this study (task, knowledge and social). Employees were aged above 45 years and were therefore in their mid-career (45–55) and late career (56 and beyond). We contacted potential participants using their organizations’ personnel records to explain the purpose of our study and to invite them to assist with our research.

Three-hundred questionnaire packages were distributed, and 207 older workers returned completed forms. Those participants, who skipped more than 5% of the responses, were excluded from the final sample (around 17% of the returned questionnaires), leaving a final sample size of 171 participants. We found that the final sample did not differ significantly in demographic terms from the participants who were excluded because of incomplete survey responses.

The final sample comprised Spanish workers aged above 45 years. The mean age was 55.7 years (SD = 4.47), 48.5% were male, and 80.3% were either married or had a stable partner. Mean job tenure was 23.7 years (SD = 11), and mean professional experience was 30,8 years (SD = 11.8). University graduates made up 75% of the population, and 86% were employed under permanent contracts, while 95.6% worked full time. Finally, 24% declared that they had no dependent persons in their care, 24.3% had one dependent, 28.4% two, and 23% had three or more dependents. This information was not provided by 13.5% of the sample.

### Measures

#### Socio-demographic data

The participants provided information about their age, gender, tenure in their organizations and educational level. Data was also collected on family structures, contract types, shiftwork and the number of dependent persons in the household.

#### Job characteristics

Morgeson and Humphrey [39] set out to develop their Work Design Questionnaire (WDQ) as a comprehensive measure. They focus on work design rather than the more restricted job design, because the concept embraces both the job and the link between jobs and the broader work context. The research undertaken by these scholars involved an exhaustive review of the literature in order to identify the principal characteristics of work and the tools applied to measure them. Based on the results obtained, they proceeded to develop the WDQ by adapting and generating the items in the questionnaire and applying them to selected samples, after which they went on to perform the pertinent psychometric reliability and validity tests (the procedure followed is described in Morgeson and Humphrey [39].

The WDQ identifies four main factors, each of which embraces various characteristics, as follows: (1) task characteristics, including autonomy (work scheduling, decision-making, and work methods), task variety, task significance, task identity, and feedback from work; (2) knowledge characteristics, comprising job complexity, information processing, problem solving, skill variety, and specialization; (3) social characteristics, including social support, interdependence (initiated and received), interaction outside the organization, and providing feedback to others; and (4) work context, consisting of ergonomics, physical demands, work conditions, and equipment use.

Based on the results of the studies published to date, the WDQ appears to provide a general and comprehensive measure of work characteristics, which can be used by scholars and practitioners alike either to conduct basic research into the nature of work or to design and redesign jobs in organizations [2, 39]. Given the radical changes seen in the nature of both tasks and jobs over the last twenty years [35], which have become ever more cognitively, relationally and emotionally oriented, adopting a whole raft of new characteristics, demands and competences [37], the WDQ may well provide the best approach to the investigation of the work characteristics experienced by today’s workers.

We used the adapted Spanish version of Morgeson’s and Humphrey’s [39] Work Design Questionnaire (WDQ) [60] to measure task, knowledge, social and contextual characteristics. The scale contained 77 items distributed in four subscales: task (24 items), knowledge (19 items), social (18 items) and contextual (13 items). The response scale ranged from 1 (“Totally disagree”) to 5 (“Totally agree”). Examples items are: “The job provides me with significant autonomy in making decisions” (task characteristics); “The job requires me to monitor a great deal of information” (knowledge characteristics); “The job activities are greatly affected by the work of other people” (social characteristics); “A lot of time was required to learn the equipment used on the job” (context characteristics).

The questionnaire displayed high reliability values. Task characteristics had a Cronbach alpha of α = .96. Knowledge characteristics had a Cronbach alpha of α = .93. Social characteristics had a Cronbach alpha of α = .92 and Context characteristics had a Cronbach alpha of α = .93.

#### Work-related goals and motivation

We used the Motivational Trait Questionnaire (MTQ-Short form) [54, 59]. The questionnaire includes 48 items measuring three dimensions, namely Personal mastery (16 items), competitive excellence (13 items) and motivation anxiety (19 items). The response scale ranged from 1 (“Very untrue of me”) to 6 (“Very true of me”). Example items are: “When I become interested in a task, I try to learn as much about it as I can” (personal mastery); “It really upsets me when someone does something better than I do” (competitive excellence); and “When working on important tasks, I get concerned that I will make a mistake” (motivation anxiety).

The questionnaire showed high reliability values in both age groups. Personal mastery had a Cronbach alpha of α = .89. Competitive excellence had a Cronbach alpha of α = .88. Motivation anxiety had a Cronbach alpha of α = .90.

#### Psychological work ability

The study used the Work Ability Index [14] to measure work ability. A mono-item measure was obtained to assess perceived work ability compared to the psychological task demands. The item was: “Assume that your work ability at its best has a value of 10 points. How high do you assess your present work ability with regard to the psychological content of your work?” The 5-point response scale ranged from “very poor” to “very good”.

#### Job mobility intentions

We used the *job mobility* item (“I will keep on working by changing job type, even when I can already retire”) from the Retirement Intentions Scale [61]. Participants were asked to reflect on the future and to state their degree of agreement or disagreement on a five-point scale (where 1 mean “completely disagree” and 5 “completely agree”).

### Statistical analysis

The hypothesized relationships were assessed using the PROCESS macro for SPSS [62] with Model 5, which estimates the indirect effect of X (Job characteristics) on Y (Psychological Work Ability/Job mobility Intentions) through the mediator M (Motivational Orientations), with a moderating role played by W (Age) in the X → Y (Job characteristics → Psychological Work Ability/Job mobility Intentions) relationship. The moderated hypothesis is supported when the direct process varies at different values assumed by the moderating variable [63]. This procedure was based on 5000 bootstrap re-samples and estimates of the direct effect and associated confidence intervals conditional on specific levels of the moderator (Mean and +/- 1 SD from Mean). When zero is not included in the 95% bias-corrected confidence interval, it may be concluded that the parameter is significantly different from zero at *p* < .05.

As self-report questionnaires were used to collect the data at the same time from the participants in each sample, common method variance may be a concern. We used the *post hoc* Harman one-factor analysis [64] to test whether variance in the data can be largely attributed to a single factor. The *post hoc* procedure was applied by examining the results of confirmatory factor analysis, which showed that a common latent factor accounted for only .0529% of the common variance. Hence, a single factor cannot account for the variance in the data, and we therefore do not consider common method variance to be a material weakness in the datasets [65].

## Results

Before testing our model, a correlation analysis was conducted among the study variables. These results are reported in Table 1. Pearson’s correlations indicated that all significant relationships between the variables were in the expected direction.

**Table 1:** Descriptive statistics and Pearson’s correlations.

*Variables*	*M (less than 55vs*.*more than 56)*	*S*.*D*. *(less than 55vs*.*more than 56)*	*1*	*2*	*3*	*4*	*6*	*7*	*8*	*9*	*10*
1. Age (years)	51.8 vs.59.5	1.8 vs. 2.5	1	-.15	**-.31**	-.11	.09	**-.30**	.02	-.01	-.09
2. Task Characteristics	3.6 vs. 3.8	.84 vs. .60	-.01	1	**-.31**	**.27**	**-.42**	**.36**	.12	**.43**	**.32**
3. Knowledge Characteristics	3.7 vs. 3.9	.72 vs. .54	-.02	**.67**	1	.14	-.15	**.51**	.08	.*28*	.19
4. Social Characteristics	3.3 vs. 3.4	.73 vs. .55	-.14	**.68**	**.54**	1	.03	.*21*	.14	-.03	.03
6. Motivation anxiety	3.3 vs. 3.4	.58 vs. .71	-.03	-.13	.01	-.07	1	-.09	.19	*-*.*24*	**-.41**
7. Personal mastery	4.7 vs. 4.5	.80 vs. .66	-.16	**.29**	.*24*	.*25*	**-.34**	1	**.30**	**.33**	**.41**
8. Competitive excellence	2.9 vs. 2.8	.75 vs. .75	-.08	.*25*	**.41**	**.42**	-.01	**.31**	1	.*28*	.14
9. Job Mobility	2.2 vs.2.4	1.0 vs. 1.2	-.05	.*24*	.09	**.30**	*-*.*22*	**.41**	.16	1	**.36**
10. PsyWA	4.2 vs.4.3	.80 vs. .71	-.08	**.58**	**.56**	**.52**	**-.31**	**.29**	.*21*	.*23*	1

Note: Values in italics represent p minus than .05. Values in bold represent p minus than.01. PsyWA means Psychological Work Ability.

Workers aged from 45 to 55 below the diagonal. Workers aged more than 56 above the diagonal.

Table 1 presents descriptive statistics for all study variables and Pearson’s correlation coefficients in both age groups. Inter-correlations between the study variables are moderate and well below their reliabilities, supporting their discriminant validity.

### Moderation analysis

The first analysis was designed to explore the moderating influence of age on the association between task characteristics and psychological work ability and job mobility intentions. Model 5 was applied to psychological work ability first and then to job mobility qua outcome. The initial general model was significant. The main effects of both task characteristics and Age were significant, as was the interaction term, as Table 2 shown.

**Table 2:** Regression analyses for moderation of age in the relationships between work characteristics and psychological work ability.

Criterion variable: Psychological Work Ability				
Predictor Variables	b^a^	SE	95%LLCI	95%ULCI
Task Characteristics	.78**	.19	.39	1.2
Age	1.2*	.54	.15	2.3
Interaction Term Task Characteristics x Age	-.32*	.14	-.59	-.04
*R*^2^	.35**			
*F*_*(6*,*164)*_	14.7**			
Criterion variable: Psychological Work Ability				
Predictor Variables	b^a^	SE	95%LLCI	95%ULCI
Knowledge Characteristics	1.04**	.23	.58	1.50
Age	1.85	.63	.59	3.10
Interaction Term Knowledge Characteristics x Age	-.47	.16	-.79	-.16
*R*^2^	.35**			
*F*_*(6*,*164)*_	14.9**			
Criterion variable: Psychological Work Ability				
Predictor Variables	b^a^	SE	95%LLCI	95%ULCI
Social Characteristics	.97	.23	.51	1.43
Age	1.73	.53	.67	2.80
Interaction Term Social Characteristics x Age	-.49	.15	-.81	-.18
*R*^2^	.32**			
*F*_*(6*,*164)*_	12.7**			

Note: N = 175.

^a^ Unstandardized regression coefficients B

*p < .05

**p < .01.

**Fig 2:**
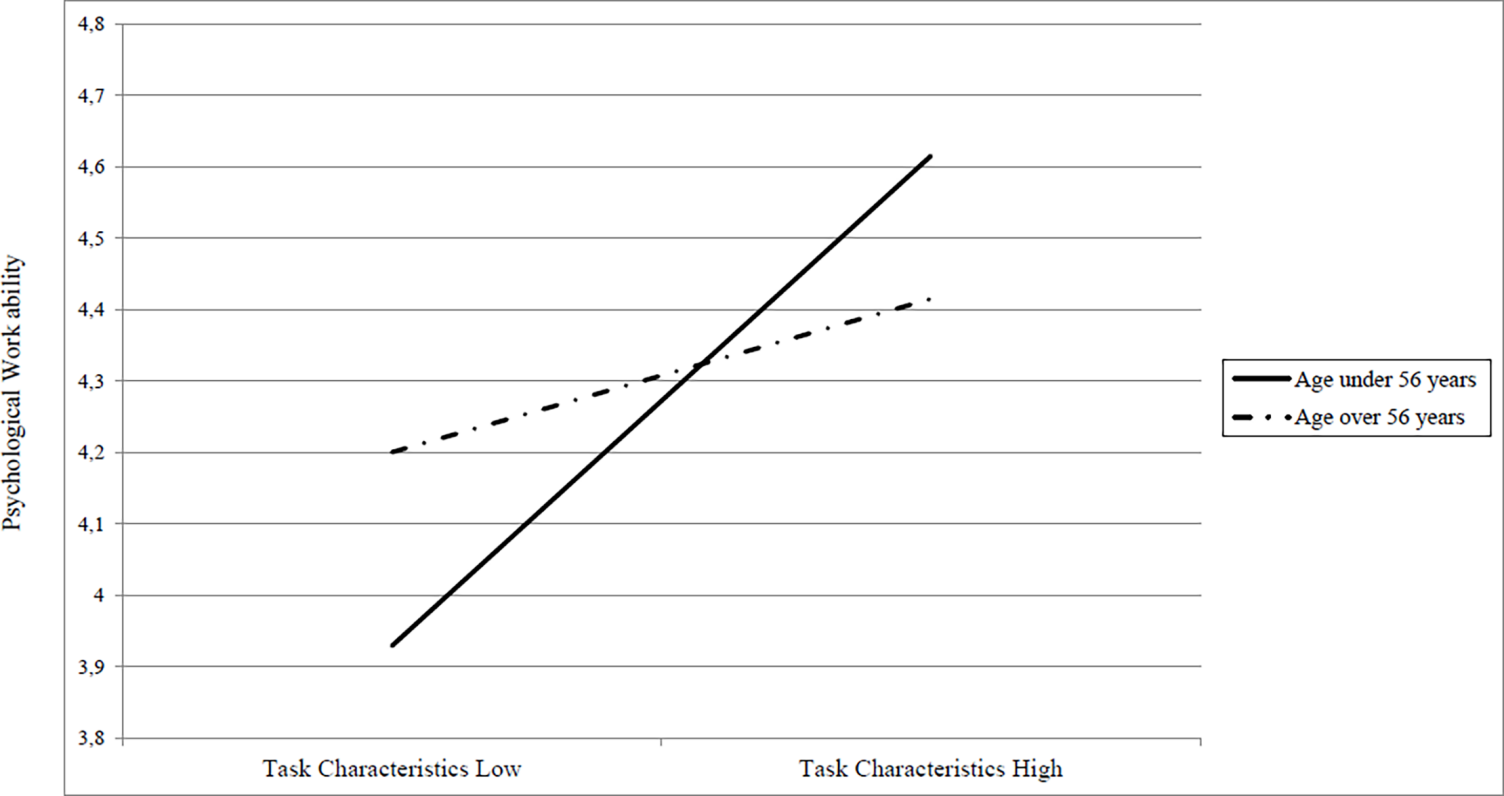
Two-way interaction between task characteristics and age in predicting psychological work ability.

Specifically, results indicated that the association between task characteristics and psychological work ability decreased in magnitude with age as Fig 2 displays, supporting hypothesis 1a. Consistent with our expectations, employees in their middle careers perceive a greater level of psychological work ability where the job features strong task characteristics such as autonomy, variety, significance and feedback from the job.

The second analysis considers the moderating effect of age on the relationships between knowledge characteristics and psychological work ability. The initial analysis of psychological work ability was significant. The main effects of both knowledge characteristics were significant, as was the interaction term, as Table 2 shown.

**Fig 3:**
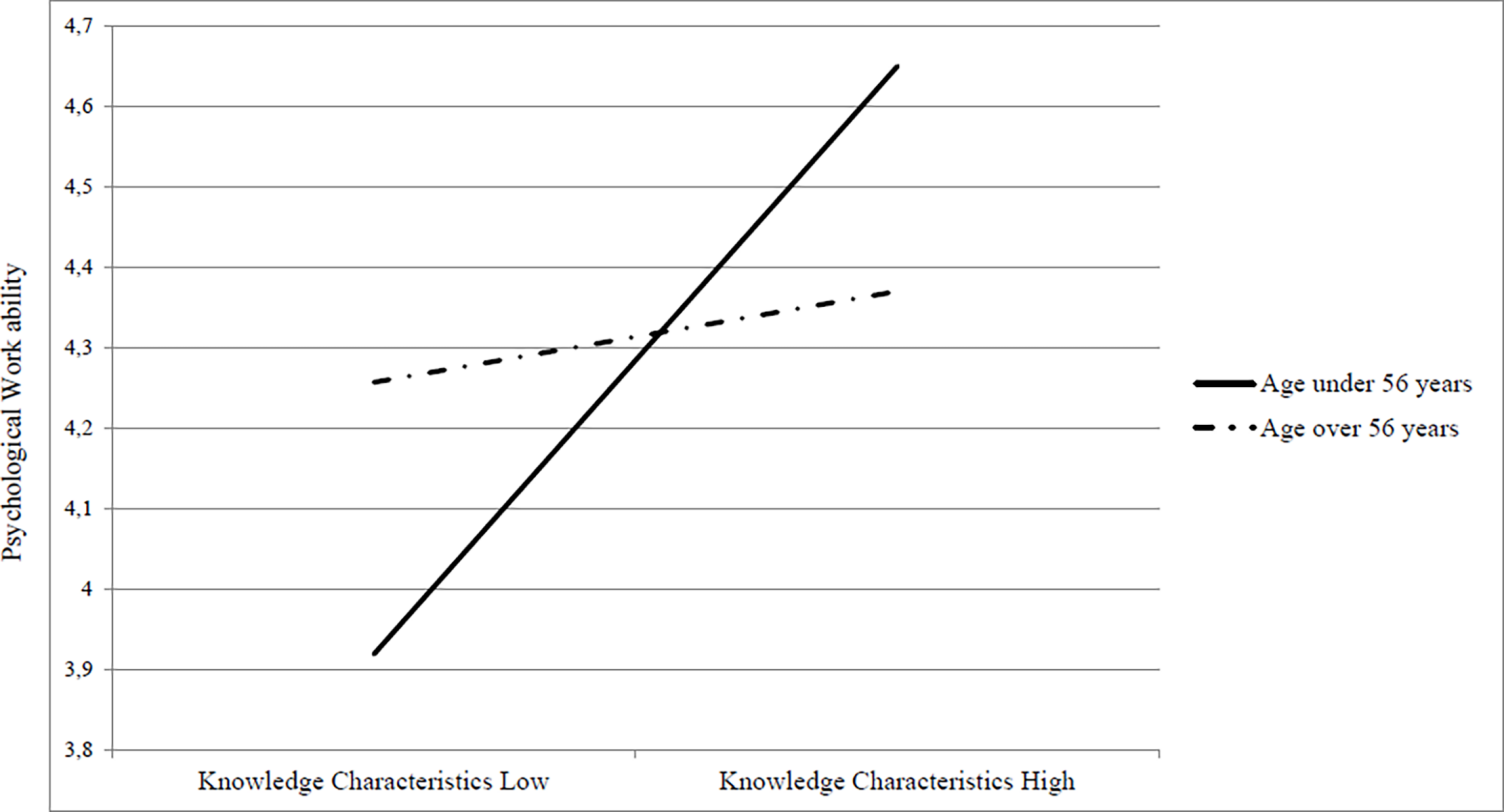
Two-way interaction between knowledge characteristics and age in predicting psychological work ability.

Specifically, results indicated that the association between knowledge characteristics and psychological work ability decreased in magnitude with age as Fig 3 displays, supporting hypothesis 1b.

The third analysis was explored the moderating effect of age on the association between social characteristics and psychological work ability. The first general model of psychological work ability was significant. The main effects of both social characteristics and age were significant, as was the interaction term, as Table 2 shown.

**Fig 4:**
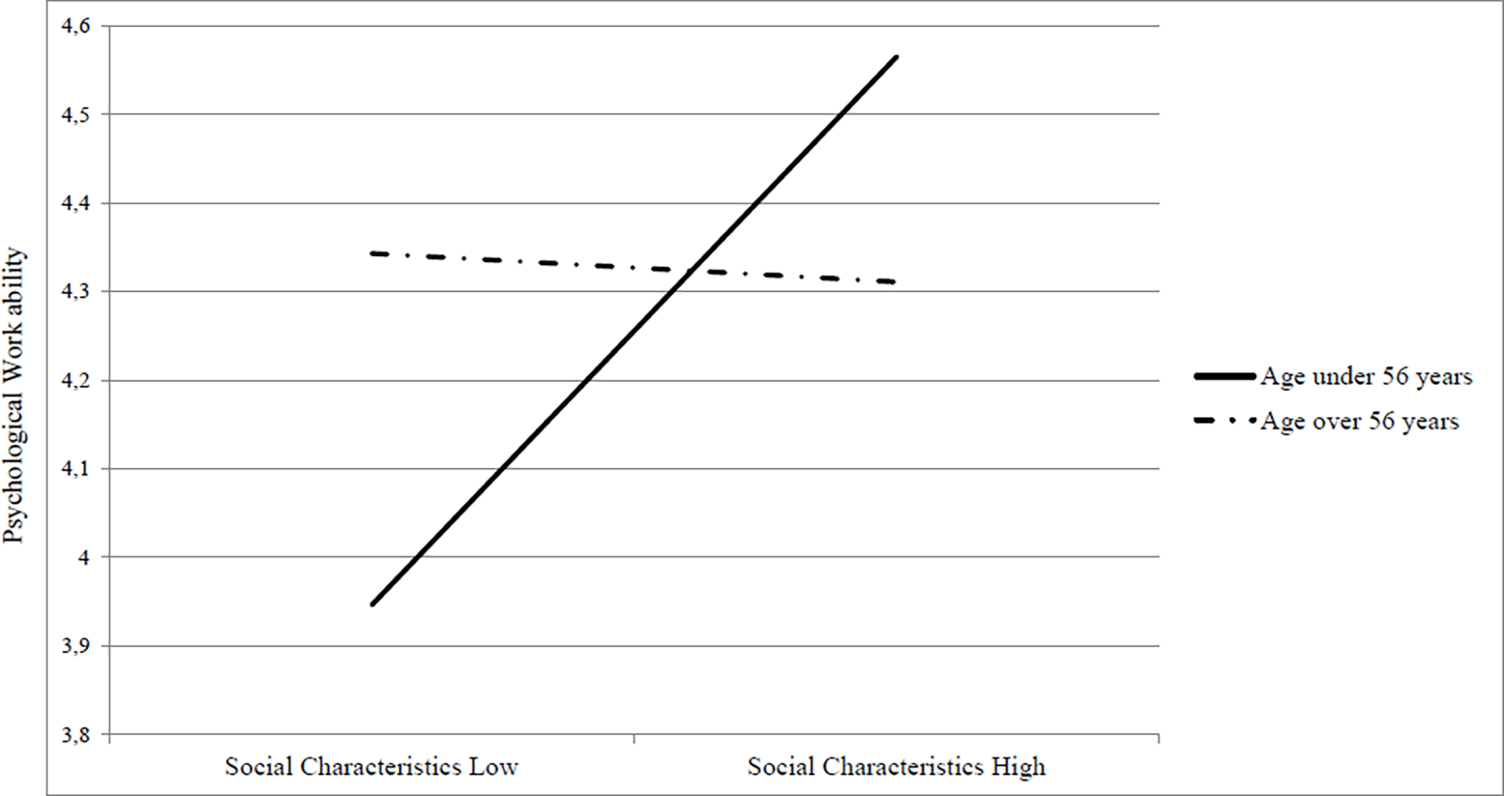
Two-way interaction between social characteristics and age in predicting psychological work ability.

Moreover, results indicated that the association between social characteristics and psychological work ability decreased in magnitude with age as Fig 4 displays, supporting hypothesis 1c.

With regard to hypothesis 2, we applied Model 5 considering job mobility Intentions qua outcome. When testing the predictive power of both task characteristics and age on Job mobility intentions, the general model was significant. The main effects of both task characteristics and age were not significant, and nor was the interaction term, contrary to hypothesis 2a, as Table 3 shown.

**Table 3:** Regression analyses for moderation of age in the relationships between work characteristics and job mobility intentions.

Criterion variable: Job mobility intentions				
Predictor Variables	b^a^	SE	95%LLCI	95%ULCI
Task Characteristics	-.23	.43	-1.09	.61
Age	-.43	1.17	-2.8	1.9
Interaction Term Task Characteristics x Age	-.21	.30	-.39	.82
*R*^2^	.19**			
*F*_*(6*,*158)*_	5.97**			
Criterion variable: Job mobility intentions				
Predictor Variables	b^a^	SE	95%LLCI	95%ULCI
Knowledge Characteristics	-.84*	.50	-1.08	.15
Age	-2.27	1.37	-4.9	.45
Interaction Term Knowledge Characteristics x Age	.68	.35	-.007	1.38
*R*^2^	.20**			
*F*_*(6*,*158)*_	6.67**			
Criterion variable: Job mobility intentions				
Predictor Variables	b^a^	SE	95%LLCI	95%ULCI
Social Characteristics	.67	.37	-.07	1.4
Age	1.8*	.88	.11	3.6
Interaction Term Social Characteristics x Age	-.45	.25	-.96	.05
*R*^2^	.21**			
*F*_*(6*,*164)*_	6.8**			

Note: N = 175.

^a^ Unstandardized regression coefficients B

*p < .05.

**p < .01.

Even though the findings for Model 5 on job mobility intentions were significant, the main effect of knowledge characteristics exhibited limited significance. Age was not significant and nor was the interaction term, contrary to Hypothesis 2b, as Table 3 shown.

Finally, when social characteristics and age have been predictors, and job mobility intention the outcome, the general model was significant. The main effect of social characteristics displayed limited statistical significance, while age was significant and the interaction term only reached a marginal effect (*p*< .10), partially supporting hypothesis 2c, as Table 3 shown.

### Simple mediation analysis

The second set of analyses was aimed to assess the indirect effect of X (job characteristics) on Y (psychological work ability first and then on job mobility intentions) through the mediator M (motivational orientations).

To begin with, results indicated a total indirect effect of task characteristics on psychological work ability, as well as significant indirect effects through motivation anxiety and personal mastery. However, the indirect effect through competitive excellence was below the threshold of statistical significance. Subsequent Sobel tests supported this result for both motivation anxiety and personal mastery. Taken together, these results point to a significant mediating effect of motivation anxiety and personal mastery in the relationship between task characteristics and psychological work ability, partially supporting hypothesis 3a, as Table 4 shown.

**Table 4:** Indirect effect of job characteristics on psychological work ability through motivational orientations.

Criterion variable: Psychological Work Ability				
Predictor variable: Task Characteristics	b^a^	SE	95% LLCI	95% ULCI
Total Indirect effect	.13	.04	.06	.24
Motivation Anxiety	.06	.03	.01	.14
Personal Mastery	.05	.02	.01	.13
Competitive Excellence	.01	.01	-.006	.05
Normal theory tests for specific indirect effects				
	b^a^	SE	Z	p
Motivation Anxiety	.06	.02	2.42	.01
Personal Mastery	.05	.02	2.13	.03
Competitive Excellence	.01	.01	.93	.35
Criterion variable: Psychological Work Ability				
Predictor variable: Knowledge Characteristics	b^a^	SE	95% LLCI	95% ULCI
Total Indirect effect	.12	.05	.01	.25
Motivation Anxiety	.02	.02	-.02	.10
Personal Mastery	.08	.04	.01	.19
Competitive Excellence	.008	.02	-.03	.05
Normal theory tests for specific indirect effects				
	b^a^	SE	Z	p
Motivation Anxiety	.02	.02	.88	.37
Personal Mastery	.08	.03	2.24	.02
Competitive Excellence	.008	.02	.37	.70
Criterion variable: Psychological Work Ability				
Predictor variable: Social Characteristics	b^a^	SE	95% LLCI	95% ULCI
Total Indirect effect	.08	.04	-.01	.17
Motivation Anxiety	.008	.03	-.05	-07
Personal Mastery	.06	.02	.01	.13
Competitive Excellence	.01	.02	-.03	.06
Normal theory tests for specific indirect effects				
	b^a^	SE	Z	p
Motivation Anxiety	.008	.02	.30	.76
Personal Mastery	.06	.02	2.08	.03
Competitive Excellence	.01	.02	.49	.61

Note: N = 175.

^a^ Unstandardized regression coefficients B.

*p < .05.

**p < .01.

***p < .001.

Secondly, related to knowledge characteristics, results indicated a total indirect effect of knowledge characteristics on psychological work ability, and significant indirect effects through personal mastery, while the indirect effects through motivation anxiety and competitive excellence do not reach statistical significance. Subsequent Sobel tests supported this result, as Table 4 shown. Taken together, these results indicated a significant mediating effect of personal mastery in the relationship between knowledge characteristics and psychological work ability, partially supporting hypothesis 3b. Thirdly, related to Social characteristics, results indicated a total indirect effect of Social characteristics on psychological work ability, and significant indirect effects through personal mastery, while the indirect effects through motivation anxiety and competitive excellence do not reach statistical significance. Subsequent Sobel tests supported this result for personal mastery, as Table 4 shown. Taken together, these results indicated a significant mediating effect of personal mastery in the relationship between social characteristics and psychological work ability, partially supporting hypothesis 3c.

Regarding hypothesis 4, we take into account the mediator role of motivational orientations in the relationships between Job characteristics and job mobility intentions as outcome. Firstly, results indicated a total indirect effect of task characteristics on job mobility intentions), and significant indirect effect through personal mastery, while the indirect effect through motivation anxiety and competitive excellence do not reach statistical significance. Subsequent Sobel tests supported this result for personal mastery, as Table 5 shown.

**Table 5:** Indirect effect of job characteristics on job mobility intentions through motivational orientations.

Criterion variable: Job Mobility Intentions				
Predictor variable: Task Characteristics	b^a^	SE	95% LLCI	95% ULCI
Total Indirect effect	.18	.06	.08	.33
Motivation Anxiety	.04	.03	-.006	.14
Personal Mastery	.10	.04	.03	.23
Competitive Excellence	.03	.02	-.006	.15
Normal theory tests for specific indirect effects				
	b^a^	SE	Z	p
Motivation Anxiety	.04	.03	1.37	.17
Personal Mastery	.10	.04	2.29	.02
Competitive Excellence	.03	.02	1.31	.19
Criterion variable: Job Mobility Intentions				
Predictor variable: Knowledge Characteristics	b^a^	SE	95% LLCI	95% ULCI
Total Indirect effect	.31	.09	.13	.52
Motivation Anxiety	.02	.03	-.01	.13
Personal Mastery	.24	.08	.13	.53
Competitive Excellence	.04	.05	-.04	.17
Normal theory tests for specific indirect effects				
	b^a^	SE	Z	p
Motivation Anxiety	.02	.03	.78	.43
Personal Mastery	.24	.08	2.74	.00
Competitive Excellence	.04	.05	.94	.34
Criterion variable: Job Mobility Intentions				
Predictor variable: Social Characteristics	b^a^	SE	95% LLCI	95% ULCI
Total Indirect effect	.18	.07	.04	.34
Motivation Anxiety	.008	.02	-.04	.08
Personal Mastery	.11	.05	.02	.25
Competitive Excellence	.06	.04	-.01	.18
Normal theory tests for specific indirect effects				
	b^a^	SE	Z	p
Motivation Anxiety	.008	.02	.33	.73
Personal Mastery	.11	.05	2.17	.02
Competitive Excellence	.06	.04	1.40	.15

Note: N = 175.

^a^ Unstandardized regression coefficients B.

*p < .05.

**p < .01.

***p < .001.

Taken together, these results indicated a significant mediating effect of personal mastery in the relationship between task characteristics and job mobility intentions, partially supporting hypothesis 4a.

Secondly, results indicated a general indirect effect of knowledge characteristics on job mobility intentions, and a significant indirect effect through personal mastery. However, the indirect effect through motivation anxiety and competitive excellence fell short of statistical significance. Subsequent Sobel tests supported this result for personal mastery. Taken together, these results indicated a significant mediating effect of personal mastery in the relationship between knowledge characteristics and job mobility Intentions, partially supporting hypothesis 4b, as Table 5 shown.

Finally, results indicated a general indirect effect of social characteristics on job mobility intentions, and a significant indirect effect through personal mastery, while the indirect effect through motivation anxiety and competitive excellence was below the statistical significance threshold. Subsequent Sobel tests supported this result for personal mastery. Taken together, these results indicate a significant mediating effect of personal mastery in the relationship between social characteristics and job mobility intentions, partially supporting hypothesis 4c, as Table 5 shown.

## Discussion

The three main objectives of this study were first to improve our understanding of the different dimensions of job characteristics (task-related, social, and knowledge-based characteristics) and their relationships with psychological perceptions of work ability and late job mobility intentions; to explore the moderating role of workers’ age on the direct relationships between job characteristics and outcomes; and finally, to explore the mediating role of older workers’ motivational orientations in the relationships between job characteristics and outcomes. Our aim, then, was to shed light on the job characteristics and motivational factors affecting workers who have entered the middle (45–55) and later (56 and over) stages of their professional lives. Our results confirm that knowledge characteristics are the most relevant factors in perceptions of psychological work ability among older workers. Both age groups display a very marked personal mastery trait, which mediates the relationships between job characteristics and both psychological work ability and late job mobility intentions.

As Wang, Olson & Shultz [47] have argued, the findings obtained in partial studies of these variables suggest divergences between workers at different moments in their working lives. It is not only that people differ in their motives for work, personal resources and perceptions at the start and end of their careers; there is also strong evidence that not all older workers are the same [7, 66]. Furthermore, the psychological aging experience is a subjective process based on an individual’s evaluations of his/her own ongoing aging process [67], which may comprise a wide range of disparate outcomes in different people.

Turning to job characteristics, it is interesting to observe that age successfully moderates the relationship between tasks, knowledge, and social characteristics and psychological work ability, which is consistent with the premise that task issues play a crucial role in maintaining older workers’ perceptions of work ability [5] and occupational well-being [68]. Thus, perceptions of work ability among the group of mid-career workers today are related with positive perceptions of task characteristics (task identity, autonomy, task significance, etc.). These results tie in with the findings of Van den Berg, Elders, de Zwart, and Burdorf [69], who reported a positive association between lack of autonomy and lower work ability, the results obtained by Finne, Christensen, and Knardahl [70], who showed that decision control was a predictor of positive outcomes at work (positive affect or mental resources measured via Work Ability Index) at the individual level, and the results reported by Weigl, Müeller, Hornung, Zacher, and Angerer [71], who noted that the use of successful aging strategies (i.e., selection, optimization, and compensation strategies) and enhanced control at work are conducive to maintaining the work ability of aging employees. This suggests that such middle-aged and older people see work as a challenge, which demands complex cognitive activity and requires specialization in a range of essential tasks.

More specifically, task characteristics (e.g., decision-making autonomy, task identity and significance) and, secondarily, knowledge characteristics (complexity, information processing, problem solving, skill variety, etc.) are the most relevant factors in perceptions of work ability in both age groups, and in perceptions of psychological work ability among workers aged between 45 and 55 years. These results suggest that the motivational aspects of work according to the classical work design model [38] remain enormously important to workers in the mid and later career stages, providing further support for the core role assigned to these factors in the literature [72]. Overall, these results are consistent with the arguments advanced by Dierdorff and Morgeson [73] on links between task characteristics, achievement and independence (occupational values) and reinforcement from the self (occupational value domain) and with Truxillo et al. [40], who posit that task, knowledge, and social work characteristics (such as job autonomy, task significance, skill variety, among others) are strongly and positively related to key indicators of occupational well-being among older workers (i.e. job satisfaction, engagement). They are also in line with the meta-analytic study performed by Ng and Feldman [74], which found stronger relationships linking job autonomy to job self-efficacy and job performance among older workers than among their younger colleagues.

The social characteristics of work were found to be significant in predicting psychological work ability but displayed only limited significance when job mobility intentions were considered qua outcome. However, it may be that social characteristics are perceived as key job features because the work performed by the multi-professional sample utilized in the study includes tasks that imply relations with other people. Nonetheless, we may recall the long-running debate over the greater or lesser degree of convergence between self-reported, subjective characteristics, as in the case of the WDQ, and objective characteristics [75], as this is a matter which could introduce bias [76]. As Hackman and Lawler [77] observed, perceptions are causal, affecting the reactions of workers towards their jobs, and measures may therefore be significant if the object of study also consists of perceptions of work ability and personal resources. Future research should explore the possible relationships between the social characteristics of work and work ability in depth, given that most published studies have tended to concentrate on task characteristics and physical and cognitive demands (e.g. [22, 78, 79].

Overall, our findings with regard to work characteristics and their relationship with the perceptions of employees in the mid and late stages of their careers may help enrich the integrated work design model proposed by Morgeson et al. [72], establishing motivational orientations as a mediating mechanism (linked to age) between work characteristics and attitudinal, behavioral, cognitive and occupational well-being outcomes, as well as psychological adjustment of older workers.

Existing studies of motivational factors have demonstrated the existence of changes brought on by age [10, 11]. Older workers tend to value extrinsic factors like status less, and intrinsic factors like independence, self-perceived performance and task autonomy more [12]. The available evidence thus points to the conclusion that there is a *“shift in people's motives rather than a general decline in motivation with age”* [80]. Our findings highlight several interesting points concerning the mediating role of work motivation in the relationships between predictors and outcomes. One similarity of all workers over the age of 45 years is the unimportance of the competitive excellence trait (other referenced goals and competition seeking), which seems to show that such motivations are not generally a factor in work ability perceptions among workers in the mid and late stages of their careers.

One of the main findings of our study was that the motivational orientation of personal mastery (desire to learn and mastery goals) successfully mediates the relationships between job characteristics and both psychological work ability and job mobility intentions. This contradicts the stereotype of a decline in interest and learning among older workers [57], while underscoring the scant motivation such people obtain from comparison and outperforming others. In the case of the study participants, workers in their late career in fact displayed this motivational orientation more strongly than those in their mid career, which suggests that the motivation of older workers is actually a much more complex matter than the impression portrayed by the usual stereotypes and hackneyed clichés.

Motivation anxiety (worry and negative emotionality) only exerted a mediating role between task characteristics and psychological work ability. These results are in line with the postulates of Kanfer and Ackerman [57], who suggest that differences appear in workers’ motivations and goals in the mid and late stages of their working lives, and these differences are more varied and complex than intuitive perceptions would suggest.

Our results support Kanfer et al. [11], who argue that the motivation of older workers is produced by the interactions of personal factors with the context, defined as person-context transaction variables. Hence, changes in motivation in mid and late career “*pertain mainly to the impact of age-related changes in competencies and motives in motivational-processing components (….)*, *and age-related changes may enhance*, *decrease*, *or have little effect on work motivation*, *depending on work circumstances*” (p. 455) [57]. In our study, work characteristics are related with certain motivational dimensions associated with perceptions about psychological work ability among mid- and late-career workers. This conclusion also provides support for the idea that there are no mono-causal links between age and work ability [5, 20, 56]. It also fits with the idea of differentiating concepts of “age” above and beyond merely chronological considerations [81, 82], distinguishing between functional age, psychosocial age, organizational age, and lifespan. Future research should take account of these differentiated age constructs, exploring their interactions with work ability and the motivational orientations of older workers. In particular, lifespan thinking is a very useful conceptual framework within which we to think about and understand motivation in aged workers, as well as dynamics in human development [83].

### Practical implications

Our results may be relevant for the adoption of retention strategies aimed at older workers, because the perception of desirable work characteristics interacting with their own motivations in turn enhances their psychological work ability, while affecting job mobility intentions. In this regard, recent data show that targeted HRM practices enhance job performance and affective organizational commitment [84], increasing employability and labor market participation among older workers [85, 86], and curbing the intention to take early retirement [87, 88] while increasing the acceptance of bridge employment [89].

In the second place, the inclusion of psychological factors like those explored in this study in the policies applied in the management of mid- and late-career workers could expand the scope of such practices beyond the usual physical and financial aspects [6]. Moreover, it would help integrate the individual-level, job-level and organizational-level factors which can impact these career stages in HR management [47]. In this regard, we concur with the argument that the proper focus for HRM is to embrace the personal resources that motivate individuals in combination with the job resources provided by the organization [90], and we postulate that this approach is equally applicable to the management of older workers.

Finally, our results may also help change commonly held stereotypes among managers, colleagues and society in general, which paint older workers as suffering poor health and lacking motivation, self-efficacy and the desire to learn, and therefore as unproductive [91, 92]. The reality is that research findings very often do not warrant these views [93]. Health, work ability and functioning do not inevitably decline in people’s middle and later working years. Training programs and interventions to reduce work hazards and promote health-conscious behaviors can prevent or mitigate age-related changes [83]. Organizations can, then, conserve explicit and implicit knowledge and foster continued competence among aging workers by providing updating opportunities, challenging task assignments and interactions with co-workers and management [83, 94]. They should therefore also strive to prevent, or at least lessen, discrimination against older workers in recruitment processes, training processes, performance assessments, career development options and so forth [5, 95, 96].

### Study limitations and future research

Despite the contributions made, this study suffers from a number of limitations. First, we have compared two age groups made up of people in their mid to late working lives. In order to explore in depth the factors influencing these career stages, however, it will be necessary to track individuals for years, examining the changes taking place in their motivation and personal resources over time [11]. In this light, it will be crucial to plan and perform extended longitudinal studies.

In the second place, we believe our measure was successful in capturing psychological work ability and job mobility intentions despite the use of self-reported measures, which implies a potential limitation of our results. Moreover, objective measures (e.g. absenteeism, health and performance), as well as the perceptions and opinions of colleagues, supervisors and managers [76], will also be needed to establish whether older workers’ perceptions are in line with actual outcomes and with the appraisals made by other organizational agents.

Third, the participants in our study were skilled professionals working in healthcare and the provision of other expert services, and the results obtained therefore cannot be generalized to work characteristics in occupations involving greater physical or other demands. Hence, future studies will be needed which use samples including multiple occupations to allow more accurate mapping of the mid- and late-career characteristics of older workers.

Finally, it will be important to continue examining both age-based motivational differences and differences between cohorts and generations, and to prevent any confusion in the interpretation of results [57], an issue which has also occasionally affected research into aging and job attitudes [97]. In this regard, Rudolph and Zacher [98] have recently proposed as an alternative to the conventional notion of generations at work that “generations are better understood from a contextualized lifespan framework that accounts for time period and history-graded developmental influences that may impact individuals’ attitudes, values, beliefs, motives, and behavior at work” (p. 113). Since the retention and management of ever larger cohorts older workers will grow increasingly important over the coming decades, it will be essential for organizations to understand and create the right conditions to foster motivation, the development of personal resources, performance, and the well-being and health of employees in the later stages of their careers, to ensure both individual quality of life and organizational efficacy, an approach which provide major benefits in terms of socio-economic sustainability, particularly in a context of growing population aging [99].
